# In Vitro and In Vivo Effects of Norathyriol and Mangiferin on *α*-Glucosidase

**DOI:** 10.1155/2017/1206015

**Published:** 2017-01-10

**Authors:** Zhi-Long Shi, Yi-Dan Liu, Yun-Yun Yuan, Da Song, Mei-Feng Qi, Xu-Juan Yang, Ping Wang, Xiao-Ying Li, Jian-Hua Shang, Zhao-Xiang Yang

**Affiliations:** ^1^Yunnan University of Traditional Chinese Medicine, Kunming 650500, China; ^2^Institute of Drug Discovery & Development, Kunming Pharmaceutical Corporation, Kunming 650106, China

## Abstract

Norathyriol is a metabolite of mangiferin. Mangiferin has been reported to inhibit *α*-glucosidase. To the best of our knowledge, no study has been conducted to determine or compare those two compounds on inhibiting *α*-glucosidase in vitro and in vivo by far. In this study, we determined the inhibitory activity of norathyriol and mangiferin on *α*-glucosidase in vitro and evaluated their antidiabetic effect in diabetic mice. The results showed that norathyriol inhibited *α*-glucosidase in a noncompetitive manner with an IC_50_ value of 3.12 *μ*M, which is more potent than mangiferin (IC_50_ = 358.54 *μ*M) and positive drug acarbose (IC_50_ = 479.2 *μ*M) in the zymological experiment. Both of norathyriol and mangiferin caused significant (*p* < 0.05) reduction in fasting blood glucose and the blood glucose levels at two hours after carbohydrate loading and it was interesting that mangiferin and norathyriol can make the decline of the blood glucose earlier than other groups ever including normal group in the starch tolerance test. However, norathyriol and mangiferin did not significantly influence carbohydrate absorption in the glucose tolerance test. Therefore, the antidiabetic effects of norathyriol and mangiferin might be associated with *α*-glucosidase, and norathyriol was more potent than mangiferin.

## 1. Introduction

With the development of society, the modern lifestyles have changed people's health status. Diabetes mellitus is becoming a major health problem with increased morbidity and high care cost. An estimated total of 438 million individuals worldwide would have diabetes and tend to severe diabetic complications by 2030 [1].

*α*-Glucosidases are one kind of the most important carbohydrate-hydrolyzing enzymes. These enzymes play a crucial role in a broad range of metabolic pathways, which are closely related to some modern diseases, such as lysosomal storage diseases, HIV, and metastatic cancer [2]. Among them, *α*-glucosidase is in the brush-border surface membrane of small intestinal cells capable of catalyzing carbohydrate into glucose which is absorbed into the blood [3]. Then the treatment of inhibiting *α*-glucosidase is an important way to suppress the postprandial hyperglycemia in patients with diabetes mellitus [4]. Thus, *α*-glucosidase inhibitors are considered to be an important therapeutic reagent for treating diabetes mellitus. At present, *α*-glucosidase inhibitors widely prescribed as antidiabetic agents such as acarbose, miglitol, and voglibose have shown the gastrointestinal side effects. In recent years, efforts have been gearing toward developing new *α*-glucosidase inhibitors from natural products.

**Figure 1:**
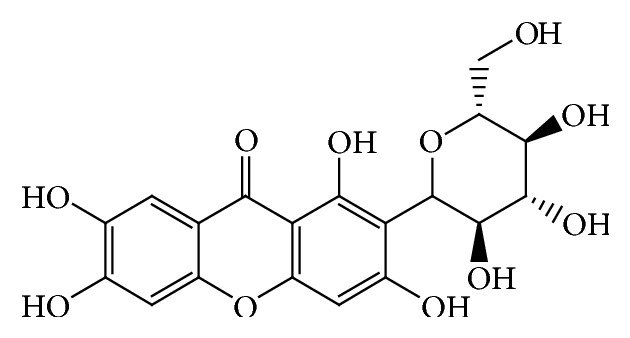
Mangiferin.

**Figure 2:**
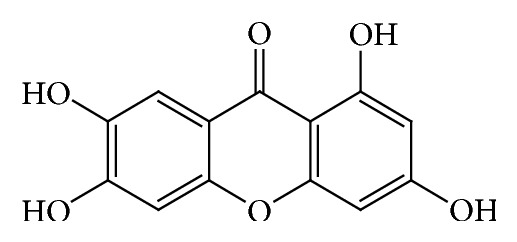
Norathyriol.

Mangiferin (Figure 1), 2-*β*-D-glucopyranosyl-1,3,6,7-tetrahydroxyxanthone, originated from* Mangifera indica *L. (Anacardiaceae), possesses a wide spectrum of pharmacological activities. Mangiferin has been reported to present with inhibitory activity of *α*-glucosidase and antidiabetic activity [5]. Norathyriol (Figure 2) is the aglycone of mangiferin. Mangiferin could be metabolized to norathyriol by intestinal bacterium [6]. There are no reports about these compounds in inhibiting *α*-glucosidase in in vitro and in vivo experiments so far. In this study, *α*-glucosidase inhibitory effects of mangiferin and norathyriol were examined in both in vitro and in vivo assays.

## 2. Materials and Methods

### 2.1. Chemicals and Reagents

*α*-Glucosidase (EC3.2.1.20) from* S. cerevisiae*, 4-nitrophenyl-*α*-D-glucopyranoside (PNP-G) as a synthetic substrate, alloxan, and dimethyl sulfoxide (DMSO) were purchased from Sigma (St. Louis, MO, USA), acarbose was biological reagent, and K_2_HPO_4_·3H_2_O, NaOH, and others were of reagent grade. Norathyriol and mangiferin were provided by Research Department of Kunming Pharmaceutical Corporation (Kunming, China).

### 2.2. Assessment of *α*-Glucosidase Inhibitory Activity

*α*-Glucosidase was assayed according to a procedure reported by Choi et al. [7] by monitoring formation of *ρ*-nitrophenol spectrometrically in a 96-well microplate at 400 nm with slight modification. Briefly, the enzyme solution (0.48 u/mL *α*-glucosidase: 50 *μ*L), phosphate buffer (100 mM pH 6.8: 150 *μ*L), and test sample in 10% DMSO (50 *μ*L) were incubated at 37°C for 10 minutes. After preincubation, 50 *μ*L of 2 mM PNP-G was added. The absorbance was recorded once per 2 minutes at 400 nm with a PowerWave XS2 microplate reader (BioTek, USA) for 10 min at 37°C. Control contained the same reaction mixture except the same volume of 10% DMSO solution instead of tested sample solution. Acarbose was dissolved in 10% DMSO and used as a positive control. All determinations were performed in triplicate. The inhibition (%) was calculated as follows: inhibition (%) = ((*V*_1_ − *V*_2_)/*V*_1_) × 100%, where *V*_1_ is the mean velocity of the control well and *V*_2_ is the mean velocity of the compounds' well; all the velocity was obtained by Gen5 2.0 (BioTek Instruments).

### 2.3. Kinetics of Enzyme Inhibition

In the kinetics measurement, inhibition assays were performed according to the above reaction conditions with inhibitors of various concentrations and the ranges of final substrate concentrations were 0.125–2.0 mM. The type of inhibition for the inhibitors was determined by double-reciprocal plot and its replot of slope versus the reciprocal of the substrate concentration.

### 2.4. Animal Experiment

Female ICR mice (20–22 g each) were obtained from the Institute of Laboratory Animal Science, Kunming Medical University, Yunnan, China. All experimental protocols were approved by the Institutional Animal Care and Use Committee following the Guide for the Care and Use of Laboratory Animals of the National Research Council. Animals were subjected to a standard pellet diet in a temperature (23 ± 2°C) and humidity (50 ± 10%) controlled facility with a 12 h light-and-dark cycle control system.

One week late, the overnight-fasted mice were i.v. injected with alloxan (60 mg/kg body weight, BW). Alloxan solution was freshly prepared in physiological saline solution and maintained on ice prior to use. The fasting blood glucose level of mice was measured 72 h after alloxan injection. Mice with >11.1 mmol/L glucose were considered hyperglycemic mice and used in the experiments. Animals were allowed free access to food and water after the alloxan injection.

### 2.5. Oral Carbohydrate Challenge Tests

The oral carbohydrate tolerance tests were carried out using starch and glucose separately. Normal mice were used as the normal control group.

#### 2.5.1. Oral Starch Tolerance Tests

Diabetic animals were randomly divided into 6 groups with 14 mice in each group. Normal control group received CMC-Na solution (concentration was 5%) only. Model group received CMC-Na solution (concentration was 5%) only. Positive group received acarbose (10 mg/kg p.o.). Norathyriol-L and norathyriol-H group were treated with norathyriol (15 mg/kg and 30 mg/kg, resp., p.o.). Mangiferin-L and mangiferin-H group were treated with mangiferin (15 mg/kg and 30 mg/kg, resp., p.o.). All the animals were treated once a day for 15 days. After the last time they received their treatment, they were fasted for four hours when they had free access to water. All animals were treated with corresponding agents orally. After 30 minutes, each mouse was given 3 g/kg of starch (p.o.). The blood was collected from the tail veins at 0, 0.5, 1, and 2 hours after starch administration, and the blood glucose concentration was measured by the blood glucose meter (GLUCOCARD II, ARKRAY, Japan) according to the instruction. Blood glucose concentrations were recorded and area under the curve (AUC) was determined. The formula for AUC determination is as follows:

(1) AUC mmol/L·h=BG0+BG0.52×0.5+BG0.5+BG12×0.5+BG1+BG22×0.5,

where BG represents the blood glucose measured at time intervals of 0, 0.5, 1, and 2 hours.

The change of blood glucose from 0.5 h to 1 h was calculated as follows:

(2) $$\begin{array}{c} \Delta {BG}_{0.5\text{–}1}={BG}_{0.5}-{BG}_{1}, \end{array}$$

where BG represents the blood glucose measure at time intervals of 0.5 hours and 1 hour.

#### 2.5.2. Oral Glucose Tolerance Test

The oral glucose tolerance test was carried out with norathyriol and mangiferin in the same way as above, but positive group received metformin (200 mg/kg, p.o.) and glucose was used here at a dose of 3 g/kg.

### 2.6. Statistical Analysis

Data are expressed as mean ± SEM. Analysis of variance (ANOVA) followed by post hoc analysis and Student's *t*-test was used for data analysis. Differences at *p* < 0.05 were considered significant.

## 3. Results and Discussion

### 3.1. *α*-Glucosidase Inhibitory Activity in In Vitro Assay

**Figure 3:**
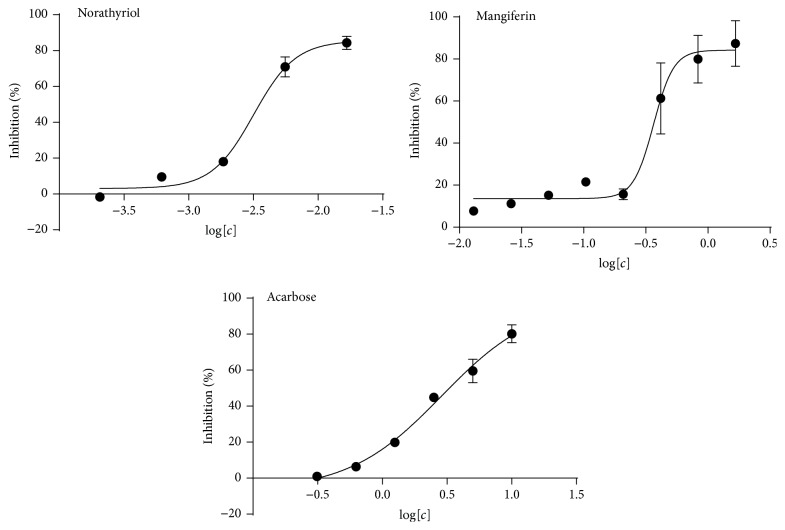
Dose response of norathyriol, mangiferin, and acarbose on inhibition for *α*-glucosidase. In the assay of dose response, norathyriol concentrations were from 0.0002 mM to 0.0167 mM, mangiferin concentrations were 0.0130–1.667 mM, and acarbose concentrations were 0.3125–10 mM. The inhibitory activity of *α*-glucosidase was evaluated at different concentrations of norathyriol (0.0002–0.0167 mM), mangiferin (0.0130–1.667 mM), and acarbose (0.3125–10 mM). In this assay, the catalytic activity of *α*-glucosidase of vehicle was served as 0% inhibitory rate.

In *α*-glucosidase inhibition assays (Figure 3), norathyriol showed the potent *α*-glucosidase inhibitory activity with an IC_50_ value of 3.12 *μ*M, while mangiferin was moderate inhibitor toward *α*-glucosidase with an IC_50_ value of 358.54 *μ*M. The positive control acarbose inhibited *α*-glucosidase with an IC_50_ value of 479.2 *μ*M, which was consistent with the published data [8].

### 3.2. Type of Inhibition

**Figure 4:**
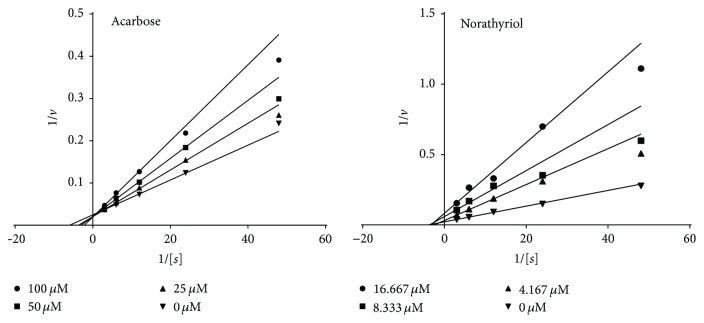
Double-reciprocal plots of the inhibition kinetics of acarbose and norathyriol toward *α*-glucosidase. In the assay, *α*-glucosidase (0.48 u/mL) was preincubated with the indicated inhibitors for 10 min at 37°C, and the reactions were initiated by adding substrate (PNP-G) in following dose from 0.33 to 0.020 mM.

In order to determine the inhibition mechanism of norathyriol, the inhibitory kinetics of the compounds at different doses to *α*-glucosidase were measured at various concentrations of substrate, and the data was exported by using the method of Lineweaver-Burk plot. The double-reciprocal plots of norathyriol (Figure 4) revealed that the reaction followed a typical noncompetitive inhibition pattern, suggesting that norathyriol is a noncompetitive *α*-glucosidase inhibitor. However, the inhibition mode of mangiferin was unable to be determined since the inhibitory activity of the compound was too weak to achieve significant signals due to the limit of equipment setting. Mangiferin was reported to inhibit *α*-glucosidase in noncompetitive mode [9], which was similar to norathyriol as determined in the current study. As a control, the inhibition pattern of acarbose was also assessed. And it was reckoned to be the competitive inhibition toward *α*-glucosidase, suggesting our assay method was valid.

### 3.3. Hypoglycemic Effect of Norathyriol and Mangiferin in Diabetic Mice

**Figure 5:**
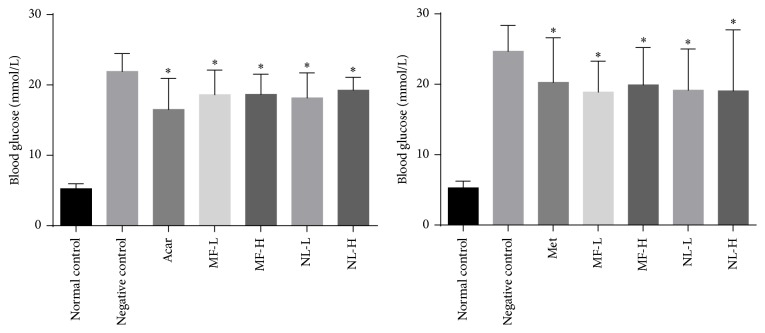
Blood glucose levels at 0 hours. Normal mice were orally given 0.5% CMC-Na solvent as normal control. Hyperglycemic mice were orally given 0.5% CMC-Na solvent (negative control), acarbose 10 mg/kg (Acar), metformin 200 mg/kg (Met), mangiferin 15 or 30 mg/kg (MF-L or H), and norathyriol 15 or 30 mg/kg (NL-L or H). Mean with asterisk (*∗*) represents significant (*p* < 0.05) difference from negative control at given time by Student's *t*-tests.

**Figure 6:**
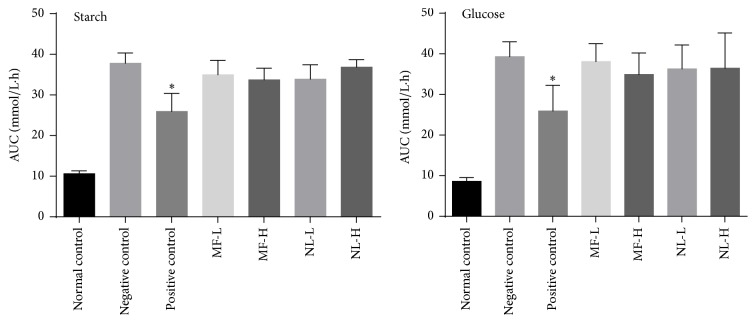
Effects of treatments on AUC after starch or glucose loading. Normal control and negative control administered CMC-Na solvent; positive control: 10 mg/kg starch test used 10 mg/kg acarbose and glucose test used 200 mg/kg metformin; MF-L or MF-H: 15 or 30 mg/kg mangiferin; NL-L or H: 15 or 30 mg/kg norathyriol. Starch was used at 3 g/kg body weight. Glucose was used at 3 g/kg body weight. ^*∗*^*p* < 0.05 compared with the negative control. One-way ANOVA followed by Dunnett's test for post hoc analysis.

**Figure 7:**
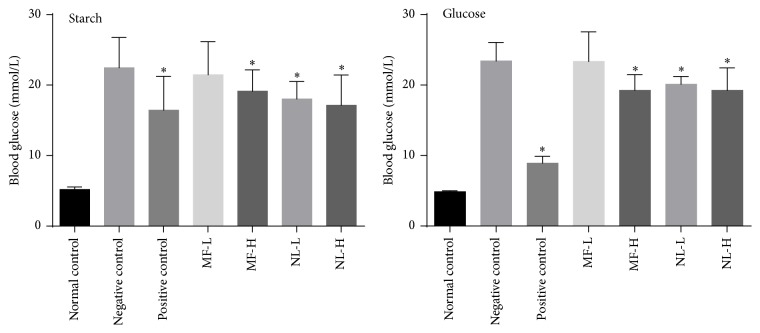
Blood glucose level at 2 hours in carbohydrate tolerance test. Hyperglycemic mice were given negative control (0.5% CMC-Na solvent), positive control (acarbose 10 mg/kg in starch test; metformin 200 mg/kg in glucose test) MF-L or H group (mangiferin 15 or 30 mg/kg), and NL-L or H (norathyriol 15 or 30 mg/kg). Mean with asterisk (*∗*) represents significant (*p* < 0.05) difference from negative control at given time by *t*-tests.

*α*-Glucosidase inhibitors have been proven to be highly effective in reduction of postprandial hyperglycemia by retarding absorption of glucose. They have been widely applied in clinic for the implications of type 2 diabetes and obesity [10]. In order to assess the therapeutic efficacy of the norathyriol and mangiferin, the inhibitory activity of the compounds was evaluated in diabetic mice. The diabetic mice were fed by starch and glucose, and the fasting blood glucose levels of the tested mice were quantitated. The mice treated with the drugs showed significantly low fasting blood glucose levels compared to negative control group (*p* < 0.05) (Figure 5). Norathyriol and mangiferin did not show obvious suppressed postprandial hyperglycemia in oral carbohydrate test (Figure 6). But norathyriol group and high dose mangiferin group reduced significantly the blood level at two-hour oral starch and glucose test (Figure 7). And the change of blood glucose from 0.5 hours to 1 hour (Table 1) showed that norathyriol group (both high dose and low dose) and mangiferin high dose group can make the glucose level of plasma decline earlier than other treatment groups in oral starch tolerant test. However, because of the difference of absorption and metabolism in monosaccharides, those phenomena were not observed in glucose loading test. This result indicates that norathyriol and mangiferin can influence absorption and metabolism of starch. And the mechanism of this effect might have relation to inhibiting *α*-glycosides.

### 3.4. Discussion

The inhibitor of *α*-glucosidase could inhibit the activity of glucosidases in the small intestine which cleave the glycosidic bonds in carbohydrate so that it reduces the glucose release from food. The inhibitors researched were classified into sugar-mimicking and nonsugar types according to their chemical structure. The clinical drugs with *α*-glucosidase inhibition such as acarbose, miglitol, and voglibose all belong to sugar-mimicking type. However, the nonsugar inhibitors of *α*-glucosidase have drawn the attention of researcher because of the limitations of sugar-mimicking inhibitors. Norathyriol and mangiferin have the same basic structure, the xanthone backbone, which is the molecular basis for *α*-glucosidase inhibition [11]. In the enzyme assays, norathyriol and mangiferin showed more potent inhibition of *α*-glucosidase than positive control (acarbose). Our study indicated that norathyriol was about 159-fold stronger than acarbose in inhibiting *α*-glucosidase. In addition, norathyriol exhibits noncompetitive mode in *α*-glucosidase inhibition, which is different from acarbose following a competitive mode.

However, the potent inhibitory activity of norathyriol and mangiferin toward *α*-glucosidase observed in in vitro assay was not in line with the responding in vivo assessment. Both of them showed mild effect in reduction of postprandial blood glucose in hyperglycemic mice models. We hypothesize that the solubility of inhibitors would affect the result of in vivo assay. Acarbose's solubility was better than norathyriol's and mangiferin's. In in vitro assay, we used dimethyl sulfoxide (DMSO) as cosolvent to dissolve the samples. But in the animal experiments, norathyriol and mangiferin could not be dissolved completely. The water-insoluble molecules would not bind with the *α*-glucosidase present in the epithelium of the small intestine.

Postprandial hyperglycemia is a characteristic and early symptom in diabetics [12]. And the high level of blood glucose would increase the risk of diabetic complication which was the major reason of disability in modern society. Although the effective forms of therapy have been applied in clinical treatment, the hypoglycemic remedy is still the primary mean for both type 1 and type 2 diabetics.

Both norathyriol and mangiferin could retard the absorption of glucose, and they could reduce significantly the fasting blood glucose level and the blood glucose level at two hours in oral carbohydrate tolerant test but they could not influence the blood glucose in oral glucose tolerant test. The results indicated that the hypoglycemic effects of these compounds are probably relevant to glucosidases.

Norathyriol and mangiferin demonstrated potent inhibition in *α*-glucosidase in vitro, and some references reported that these compounds also have many other bioactivities in diabetic pathways such as AMP-activated protein kinase [13], PPAR-*α* [14], and PTP1B [15]. Also mangiferin was found abundantly in natural source such as Rhizome of Common* Anemarrhena*,* Mangifera indica* L., and Mangosteen (*Garcinia mangostana*). Norathyriol is a natural metabolite of* Mangifera* in the human intestine, and the oral availability and safety could be much better than the novel synthesized compounds. Although the dissolvability, bioavailability, and other druggable properties of norathyriol should be improved, norathyriol and its derivate would be the promising drugs for diabetic therapy.

## Figures and Tables

**Table 1:** The change of blood glucose from 0.5 hours to 1 hour after carbohydrate loading. Normal control and negative control administered CMC-Na solvent; positive control: 10 mg/kg starch test used 10 mg/kg acarbose and glucose test used 200 mg/kg metformin; MF-L or MF-H: 15 or 30 mg/kg mangiferin; NL-L or H: 15 or 30 mg/kg norathyriol. Starch was used at 3 g/kg body weight. Glucose was used at 3 g/kg body weight. The data unit below is mmol/L·h.

	Normal	Negative	Acar	MF-L	MF-H	NL-L	NL-H
Starch	0.33	1.3	0.661538	3.683333	−0.00833	−0.26667	−0.2
Glucose	−1.23	−4.66923	−9.11429	−3.85	−2.56923	−1.88333	−3.87692

## References

[1] Shaw J. E., Sicree R. A., Zimmet P. Z. (2010). Global estimates of the prevalence of diabetes for 2010 and 2030. *Diabetes Research and Clinical Practice*.

[2] Borges de Melo E., da Silveira Gomes A., Carvalho I. (2006). *α*- and *β*-glucosidase inhibitors: chemical structure and biological activity. *Tetrahedron*.

[3] Girish T. K., Pratape V. M., Prasada Rao U. J. S. (2012). Nutrient distribution, phenolic acid composition, antioxidant and alpha-glucosidase inhibitory potentials of black gram (*Vigna mungo* L.) and its milled by-products. *Food Research International*.

[4] Casirola D. M., Ferraris R. P. (2006). *α*-glucosidase inhibitors prevent diet-induced increases in intestinal sugar transport in diabetic mice. *Metabolism*.

[5] Muruganandan S., Srinivasan K., Gupta S., Gupta P. K., Lal J. (2005). Effect of mangiferin on hyperglycemia and atherogenicity in streptozotocin diabetic rats. *Journal of Ethnopharmacology*.

[6] Sanugul K., Akao T., Li Y., Kakiuchi N., Nakamura N., Hattori M. (2005). Isolation of a human intestinal bacterium that transforms mangiferin to norathyriol and inducibility of the enzyme that cleaves a C-glucosyl bond. *Biological and Pharmaceutical Bulletin*.

[7] Choi C. W., Choi Y. H., Cha M.-R. (2010). Yeast *α*-glucosidase inhibition by isoflavones from plants of leguminosae as an in vitro alternative to acarbose. *Journal of Agricultural and Food Chemistry*.

[8] Rivera-Chávez J., González-Andrade M., Del Carmen González M., Glenn A. E., Mata R. (2013). Thielavins A, J and K: *α*-glucosidase inhibitors from MEXU 27095, an endophytic fungus from *Hintonia latiflora*. *Phytochemistry*.

[9] Wu C., Shen J., He P. (2012). The *α*-glucosidase inhibiting isoflavones isolated from belamcanda chinensis leaf extract. *Records of Natural Products*.

[10] Tundis R., Loizzo M. R., Menichini F. (2010). Natural products as *α*-amylase and *α*-Glucosidase inhibitors and their hypoglycaemic potential in the treatment of diabetes: an update. *Mini-Reviews in Medicinal Chemistry*.

[11] Liu Y., Ma L., Chen W.-H., Park H., Ke Z., Wang B. (2013). Binding mechanism and synergetic effects of xanthone derivatives as noncompetitive *α*-glucosidase inhibitors: a theoretical and experimental study. *The Journal of Physical Chemistry B*.

[12] De Veciana M., Major C. A., Morgan M. A. (1995). Postprandial versus preprandial blood glucose monitoring in women with gestational diabetes mellitus requiring insulin therapy. *New England Journal of Medicine*.

[13] Wang F., Yan J., Niu Y. (2014). Mangiferin and its aglycone, norathyriol, improve glucose metabolism by activation of AMP-activated protein kinase. *Pharmaceutical Biology*.

[14] Hsun-Wei Huang T., Peng G., Qian Li G., Yamahara J., Roufogalis B. D., Li Y. (2006). Salacia oblonga root improves postprandial hyperlipidemia and hepatic steatosis in Zucker diabetic fatty rats: activation of PPAR-*α*. *Toxicology and Applied Pharmacology*.

[15] Ding H., Zhang Y., Xu C. (2014). Norathyriol reverses obesity- and high-fat-diet-induced insulin resistance in mice through inhibition of PTP1B. *Diabetologia*.

